# Breast Implant–Associated Anaplastic Large Cell Lymphoma: A Case Report and Review of the Literature

**DOI:** 10.1155/2017/6478467

**Published:** 2017-10-31

**Authors:** Daniel E. Ezekwudo, Tolulope Ifabiyi, Bolanle Gbadamosi, Kristle Haberichter, Zhou Yu, Mitual Amin, Kenneth Shaheen, Michael Stender, Ishmael Jaiyesimi

**Affiliations:** ^1^Department of Hematology and Oncology, Oakland University William Beaumont School of Medicine, William Beaumont Hospital, 3601 W. 13 Mile Rd., Royal Oak, MI 48073, USA; ^2^Oakland University William Beaumont School of Medicine, 3601 W. 13 Mile Rd., Royal Oak, MI 48073, USA; ^3^Department of Pathology and Laboratory Medicine, Oakland University William Beaumont School of Medicine, William Beaumont Hospital, 3601 W. 13 Mile Rd., Royal Oak, MI 48073, USA

## Abstract

Breast implant–associated anaplastic large T-cell lymphoma has recently been recognized as an entity, with few reports describing the two common subtypes: in situ (indolent) and infiltrative. Recently, the infiltrative subtypes have been shown to be more aggressive requiring adjuvant chemotherapy. We report a rare case of breast implant–associated anaplastic large cell lymphoma (BIA-ALCL) in a 65-year-old Caucasian female following silicone breast implantation and multiple capsulectomies. We discuss the rare presentation of this disease, histopathologic features of the indolent and infiltrative subtypes of ALCL, and their clinical significance. We also review the literature for up-to-date information on the diagnosis and clinical management. Treatment modalities including targeted therapy are also discussed. Although BIA-ALCL is rare, it should always be considered as part of the differential diagnosis especially in women with breast implants. Given the increasing rate of breast reconstruction and cosmetic surgeries, we anticipate a continuous rise in incidence rates of this rare disease; thus, caution must be taken to avoid misdiagnosis.

## 1. Introduction

Breast lymphoma represents approximately 0.7% of all lymphomas, of which 8% are peripheral T-cell lymphomas (PTCLs) [1]. The majority of reported PTCLs are ALK-negative anaplastic large T-cell lymphomas (ALCLs). Breast implant–associated anaplastic large T-cell lymphoma (BIA-ALCL) has been reported but only recently has gained recognition as a distinct entity. Two different subtypes with a possible histogenetic relationship have been described including in situ BIA-ALCL and infiltrative BIA-ALCL; these subtypes have significantly different prognostic implications with the infiltrative subtype showing worse prognosis [2]. Generally, in situ BIA-ALCL follows an indolent clinical course after breast implant removal, whereas infiltrative BIA-ALCL is more aggressive, requiring additional therapy after implant removal [2]. Thus, accurate histopathologic diagnosis is crucial for risk assessment and therapeutic management. Recent advances in therapeutic approaches have resulted in significant improvement in the overall survival of patients with BIA-ALCL. More recently, targeted therapy utilizing the anti-CD30 antibody brentuximab-vedotin (BV) has shown promising results [3, 4].

## 2. Case Presentation

**Figure 1:**
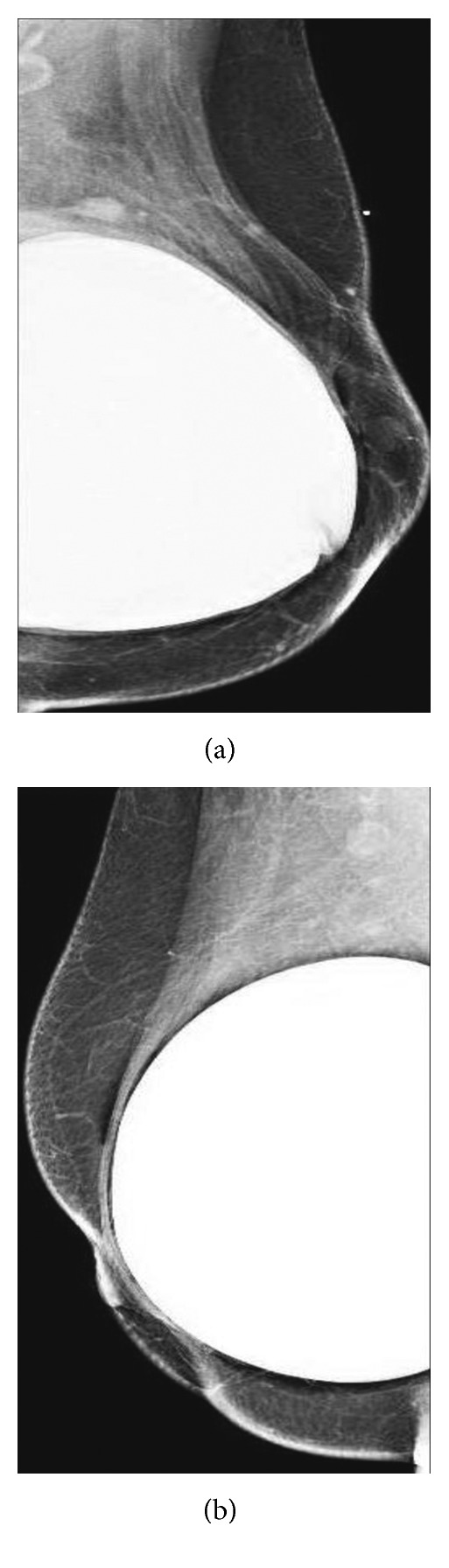
Mammogram showing silicone implant in the (a) left and (b) right breasts.

**Figure 2:**
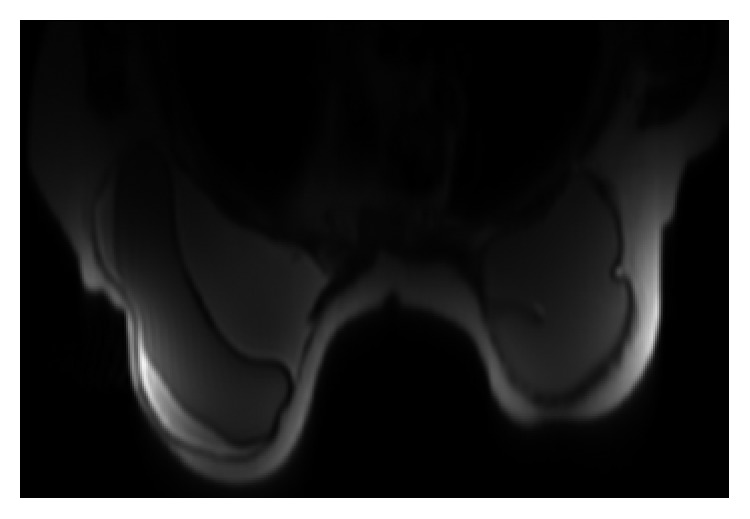
MRI showing fluid accumulation around the left breast subpectoral silicone implant.

**Figure 3:**
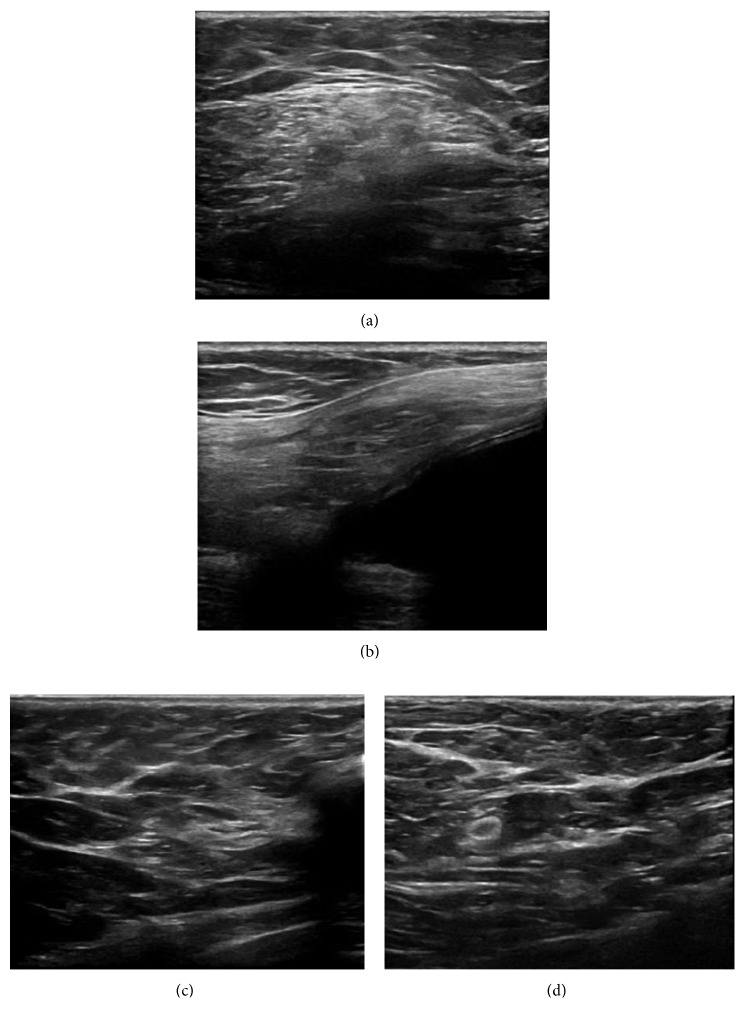
Ultrasound of the left breast ((a) transverse view, (b) sagittal view at 12 : 00 position) and the right breast ((c) transverse view, (d) sagittal view at 10 : 00 position).

A 65-year-old Caucasian female had a past medical history significant for bilateral fibrocystic breast disease resulting in bilateral subcutaneous mastectomy, followed by bilateral cosmetic breast reconstruction with textured silicone gel implants at age 30 (Figure 1). Subsequently, she had multiple complications from the implants including capsule contractures, infections, chronic seroma, and ruptured breast implants, leading to capsulectomy and implant replacements 15 and 22 years post original implantation. During these periods, the patient was noted to have several areas of calcifications in both breasts (L > R) that had been monitored with routine mammography. The patient noted that at 30 years post original implantation, her left breast became edematous; however, this self-resolved a few months later. Two years later, edema was noted again in the left breast and confirmed by MRI (Figure 2)], resulting in a third replacement of the silicone gel implant. Recently, at 35 years post original surgery, the patient presented with swelling in the left breast which progressively worsened over 2–3 months. A targeted ultrasound examination of the left breast at the approximate twelve o'clock position, left axilla, and of the right breast at the ten o'clock position over areas of concern demonstrated no discrete cystic or solid abnormalities (Figure 3). Unremarkable parenchyma was observed throughout the entire region. However, given the extent of edema in the left breast and associated pain, bilateral total capsulectomy was performed for a fourth time.

**Figure 4:**
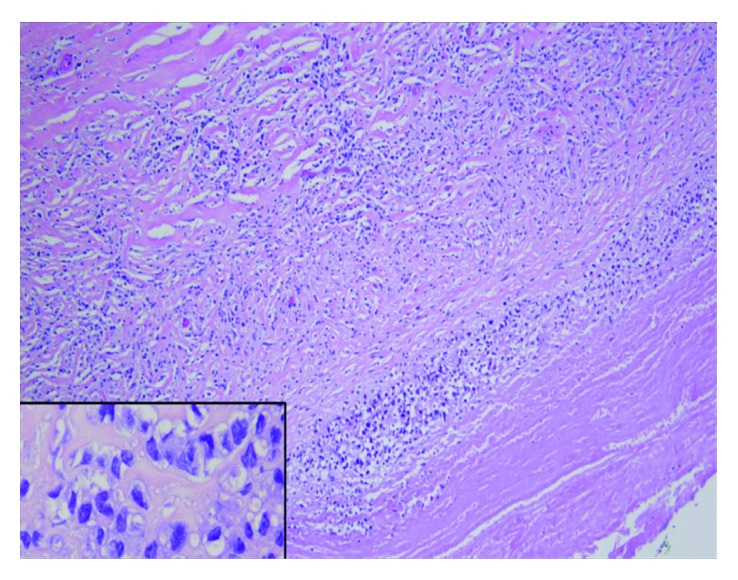
Representative sections of the left breast capsule. Larger image: the capsule contains small lymphocytes, macrophages, plasma cells, and occasional eosinophils. In addition, there are clusters of large neoplastic cells throughout the capsule, adjacent to the luminal fibrinoid necrosis (hematoxylin and eosin, 100x). Inset image: the large neoplastic cells are pleomorphic with hyperchromatic nuclei and abundant clear to slightly eosinophilic cytoplasm. Occasional “hallmark” cells with eccentric horseshoe- and kidney-shaped nuclei (hematoxylin and eosin, 1000x).

**Figure 5:**
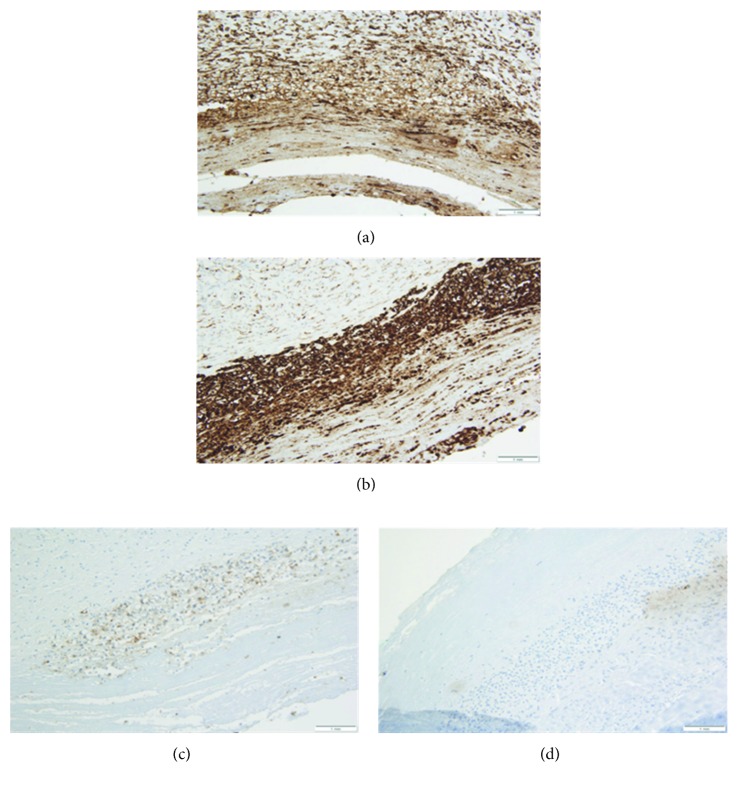
Select immunostaining of the region of capsular tissue with atypical cells. (a) Large neoplastic cells stain positive with CD43, confirming T-cell lineage (200x). (b) In addition, the neoplastic cells have uniform membranous and Golgi staining with CD30 (200x). (c) There is weak staining with epithelial membrane antigen (EMA) (200x). (d) The cells lack expression of ALK (200x).

Breast tissue obtained during surgery was sent for pathologic evaluation. The left capsulectomy specimen revealed a thickened fibrous capsule with chronic inflammation, consisting of small lymphocytes, eosinophils, plasma cells, and macrophages. The luminal surface of the breast capsule showed fibrin deposition with a thin row of highly atypical cells. The atypical cells were large and pleomorphic, with hyperchromatic nuclei and occasional prominent nucleoli noted along with abundant clear to slightly eosinophilic cytoplasm (Figure 4). Immunohistochemical analysis demonstrated strong CD30, CD43, and MUM1 expression, while EMA was weakly positive (Figure 5). The cells did not express ALK (Figure 5), CD20, CD79a, or estrogen receptor. The overall morphology and immunohistochemical profile were diagnostic for breast implant–associated anaplastic large cell lymphoma.

**Figure 6:**
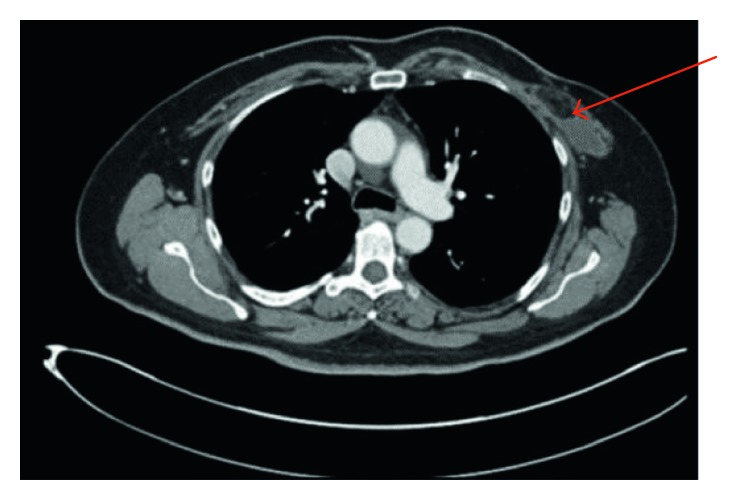
CT scan of chest: large fluid collection in the left chest wall measuring 12.9 × 2.8 × 10.1 cm.

A bone marrow evaluation, including flow cytometry studies, showed unremarkable trilineage hematopoiesis without evidence of involvement by lymphoma or metastatic malignancy. Cytogenetic examination of the bone marrow revealed twenty metaphase cells with a normal female diploid karyotype with no consistent numerical or structural chromosome aberrations. Computed tomography (CT) scans of the neck, chest, abdomen, and pelvis with IV and oral contrast were essentially negative for any malignancy or lymphadenopathy except for the noted fluid collection in the left breast measuring 12.9 × 2.8 × 10.1 cm (Figure 6). Whole body positron emission tomography–computed tomography (PET/CT) scan with fluorodeoxyglucose (FDG) radiotracer revealed increased FDG uptake along the anterior chest wall, slightly greater on the right than the left, with a maximum SUV of 4.7 and 4.1, respectively. No other area of increased FDG uptake was noted. Given the in situ subtype of ALCL noted in our patient, she underwent capsulectomy with no other local or systemic therapy. She remains clinically well after 12 months follow-up under close surveillance with our clinic.

## 3. Discussion

Non-Hodgkin lymphoma of the breast is exceedingly rare; the majority diagnosed are of B-cell origin including diffuse large B-cell lymphoma, extranodal marginal zone lymphoma, follicular lymphoma, primary effusion lymphoma, and lymphoplasmacytic lymphoma [5–7].

Peripheral T-cell lymphoma (PTCL) of the breast is less frequently reported and represents only 10% of all breast lymphomas. In breast implant patients, >90% of these are ALK-negative ALCL, compared to 37% in non–breast implant patients [1, 8]. To date, there are more than 300 reported cases of BIA-ALCL worldwide; however, only about 130 report pathologic markers, the majority of which were in the United States (67.4%) [9, 10]. The US Food and Drug Administration (FDA) estimated the incidence of BIA-ALCL to be 0.6–1.2 per 100,000, based on reported cases of BIA-ALCL among an estimated 5–10 million women with breast implants [11].

Anaplastic large T-cell lymphoma belongs to the spectrum of lymphoproliferative CD30^+^ disease and can manifest as either cutaneous or systemic disease. There are two known subtypes of ALCL of the breast: (a) in situ, in which disease proliferation is confined to the capsule and is often associated with seroma as in our patient, and (b) infiltrative, mostly associated with tumor mass with cells infiltrating the capsule and adjacent tissues [2]. Of the reported cases of BIA-ALCL, approximately 50% have seroma involvement, which is thought to be associated with a better prognosis, although some have argued a histogenetic relationship with the inflammatory subtype of BIA-ALCL [2]. The most common presenting symptom is unilateral swelling related to periprosthetic fluid collection more than a year after implantation [12, 13]. Other symptoms include pain, rash, pruritus, and capsular contracture [13, 14]. Patients present rarely with a mass that protrudes from the fibrous capsule, leading to an implant with an irregular texture [13]. BIA-ALCL has been reported in both silicone and saline (either textured or smooth) implants; for instance, out of the 359 cases of BIA-ALCL reported to the US-FDA in 2017, 28 of the cancers were in women who received breast implants with smooth surfaces, whereas 203 were in women who had breast implant version with a textured surface [15]. Inflammatory TH17 T-cells are found in greater numbers in textured compared to smooth breast implants [9]; however, no causal link between the type of implant and ALCL has been established. Chronic inflammation within the capsule is believed to be the cause of ALCL [16].

Histologically, ALCL may present as epitheloid-like, mimicking poorly differentiated breast carcinoma [12, 13]; thus, accurate immunohistochemical and histopathological evaluation is necessary. Usually, BIA-ALCL has uniform expression of CD30 with atypical cytology and is cytokeratin-negative [8]. Cells are large and pleomorphic with dispersed chromatin and multiple or single prominent nucleoli and have eosinophilic to amphophilic cytoplasm [9, 13]. Hallmark cells can occasionally be observed, with horseshoe- or kidney-shaped nuclei and a paranuclear eosinophilic region [5, 13]. Cytogenetically, about two-thirds of BIA-ALCL present with clonal rearrangement of the T-cell receptor gene [17].

Imaging of affected breasts often shows an effusion surrounding the implant with or without a mass [13]. Overall, the lymphoma cells in BIA-ALCL histologically and morphologically resemble that of ALK-negative systemic ALCL. Despite these similarities, the clinical outcome of BIA-ALCL can differ greatly from that of systemic ALCL. A recent report by Laurent et al. [2] indicates that systemic ALCL has an aggressive clinical course closer to the infiltrative subtype of BIA-ALCL than to the in situ subtype. For instance, 2-year overall survival of systemic ALCL and BIA-ALCL are 48% and 52.5%, respectively, whereas the in situ subtype has >95% survival at 2 years [12, 18].

Proposed therapeutic approaches for patients with BIA-ALCL have ranged from surgery with or without standard chemotherapy and with or without radiation to more recent targeted therapy. Gidengil et al. elaborated the use of all these treatment options in a review of 54 cases of BIA-ALCL in which 57% were treated with standard chemotherapy treatment for non-Hodgkin lymphoma including cyclophosphamide, hydroxydaunorubicin, vincristine, and prednisone (CHOP) with or without other chemotherapy agents, 48% received radiation therapy mostly to the chest wall, and 11% received stem cell transplants [14]. Agents such as etoposide have also been reported in treatment therapies for BIA-ALCL [19].

Due to the indolent course of in situ disease, capsulectomy alone without aggressive chemotherapy has been suggested as a more appropriate approach in those patients with disease confined to the capsule [9, 16]. In these patients, removal of implants and capsulectomy treatment alone have favorable outcomes, with the mean duration of remission approximately 16 months [12]. Disease recurrence has also been reported and may present as either localized or metastatic [14]. A more aggressive approach is recommended for patients with the infiltrative subtype of BIA-ALCL. Patients with positive regional lymph node involvement at diagnosis have a higher rate of recurrence, and nodal and/or systemic involvement is often the cause of death [12]. In cases of lymph node involvement with cytogenetic abnormalities, capsulectomy followed by CHOP plus etoposide upon relapse has been suggested [20]. Patients who present with a distinctive mass may have a worse prognosis as this often times indicates the infiltrative subtype of ALCL and require aggressive treatment including chemotherapy and radiation therapy [8, 13]. Other factors such as staging at the time of diagnosis should also be considered. Brody et al. reported nine deaths in patients with BIA-ALCL even after repeated therapies and noted that four out of the nine deaths presented with a mass [9].

Recently, targeted therapy has shown encouraging results. More than 90% of ALCL overexpress CD30 antigen, thus making this a favorable target for future drug designs. The approval of brentuximab-vedotin (BV), a CD30-specific monoclonal antibody conjugated to the tubulin toxin monomethyl auristatin E (MMAE) [21], provides a promising therapy for patients who do not respond to conventional chemotherapy or salvage high-dose chemotherapy and stem cell transplantation [19, 22]. The proposed mechanism of action of BV involves MMAE binding to the CD30 receptor and internalization into the cell, where it induces growth arrest and apoptosis [21]. BV was approved for treatment of Hodgkin lymphoma (HL) and ALCL unresponsive to previous treatment. Peripheral sensory neuropathy is the most common side effect, which has been shown to be dose dependent, and partially reversible following dose reduction or treatment cessation. Other side effects include nausea, fatigue, pyrexia, diarrhea, rash, constipation, and neutropenia [21]. Recent phase II trials reported overall response rates of 75% in patients with HL and 86% in patients with systemic ALCL that have relapsed or were unresponsive to previous treatments. The complete response rates were 34% and 57% for patients with HL and ALCL, respectively [22]. BV has also been shown to be effective as a first line treatment. Oregel et al. reported successful treatment of a critically ill patient with ALK-negative ALCL involving the axillary lymph nodes [23].

The lack of complete response in some patients could be due to development of resistance to BV. Loss of CD30 expression following treatment with BV has been noted in 2 cases of ALK-negative ALCL [3, 4]. Downregulation of CD30 has also been observed in resistant ALCL cell lines [22], supporting this mechanism in the development of resistance to BV. Currently, clinical trials assessing the use of BV with CHOP or CHP (CHOP without vincristine) have shown promising efficacy with tolerable toxicities in CD30^+^ PTCL [24]. Thus, BV could potentially be an option for treatment of aggressive BIA-ALCL refractory to chemotherapy or even as first line treatment.

Apart from anti-CD30 immunotherapy, emerging studies are revealing other possible targets in patients with ALCL especially those with ALK positivity. For instance, a recent study by Laimer et al. showed that high expression of platelet-derived growth factor receptor (PDGFR a/b) was observed in mouse model effected with human large T cell lymphoma. Their study revealed that combination of standard chemo/immunotherapy plus anti-PDGFR therapy such as imatinib resulted in complete remission in patients with relapsed ALCL after autologous transplantation [25]. Although still in the early stage of research, their results offer great potentials for patients with more aggressive variant of ALCL especially those with ALK expressivity. Currently, a clinical trial (a window of opportunity trial) investigating the therapeutic impact of combination treatment with anti-CD30 plus imatinib is ongoing [26].

## 4. Conclusion

BIA-ALCL is a rare breast lymphoma that has both indolent (in situ) and aggressive (infiltrative) subtypes. Tumor mass at presentation could be used a marker of the more aggressive type requiring standard chemotherapy with or without radiation plus capsulectomy. Although prognosis for patients with an in situ disease subtype is excellent, the infiltrative subtype has a prognosis similar to systemic ALCL. Early histopathologic diagnosis is crucial to initiating the right treatment course. Patients should continue close surveillance following completion of treatment to monitor for disease recurrence.
